# 

**Published:** 1879-08

**Authors:** 


### INDEX.

### A.

PAGE.

Abortion and its Treatment, . . . . 433
Abscess, Perityphlitic, by Herman Mynter, M. D., . . . . . . . . . 85
Absorbing Power of Wounds, . . . 450
Acne, Treatment of, . . . . . . 71
Ague, Nitrate of Amyl in, . . . 213
Albumen, Delicate Test for, . . . 546
Alcohol in Animal Tissues, The Presence of, . . . . . . . . . 447
Alumni Associations, . . . . . . 418
American Medical Association, . . 511
Ammonia, Intravenous Injection of, . 61
Aneurism Traumatic, Report of, by Fred. Peterson, M. D., . . . . . . 199
Ammoniacal Cystitis, Boracic Acid in, . 163
Aneurism of the Brachial Artery, by Herman Mynter, M. D., . . . . . 147
Angular Curvature of Spine Treated by the Paper-and-Glue Brace, by Bernard Bartow, M. D., . . . . . . . 202
Anus Imperforate, by Thos. Lothrop, M. D., . . . . . . . . . . 151
Anthelmintic, A New, . . . . . . 408
Antiseptic Treatment in Wounds on the Head, Mortality of, by Herman Mynter, M. D., . . . . . . . . . 159
Antiseptic Treatment in Extraction of Cataract, . . . . . . . . . 65
Antisepsis in Midwifery, by Thomas Lothrop, M. D., . . . . . . . 41
Appendix Vermiformis, Medicinal Substances Imbedded in the Walls of the, . 404
Astringents in Chronic Conjunctivitis, . 439
Atrophy of the Optic Nerve in Erysipelas of the Face, by Lucien Howe, M. D., . 207
Atropia, Treatment of Whooping Cough by, . . . . . . . . . . . 357
Atropia in Traumatic Tetanus, . . 308
Audiphone and Kindred Instruments, . 484
Auscultation in Uterine Hemorrhage, . 164

### B.

PAGE.

Balsam of Peru in Pruritis, . . . . 361
Bartlett, F. W., M.D., on Puerperal Eclampsia, . . . . . . . . . 248
Bartow, Bernard, M.D., on Angular Curvature of Spine, treated by the Paper-and-Glue Brace, . . . . . 202
Bartow, Bernard, M.D., on Hydrocele, . 527
Battery Fluid, . . . . . . . . 71
Battey's Formula Iodized Phenol—In Eczema Marginatum, . . . . . 405
Beckwith, Hon. Charles, on Testimony of Medical Experts, . . . . . 421, 469
Belladonna in Poisoning by Opium, . 264
Benzoate of Soda in Diphtheria, . . 410
Board of Health Reports, . . . . 550
Boracic Acid in Ammoniacal Crystitis, . 163
Bladder of a Child, New growth in the, by Herman Mynter, M. D., . . . . 9
Bladder, Catarrh of the, . . . . . 444
Blood, Spectrum Analysis of, by W. H. Pitt, M. D., . . . . . . . . . 1
Brachial Artery, Aneurism of the, by Herman Mynter, M. D., . . . . 147
Bromide of Ethyl, . . . . . . . 353
Bromide of Ethyl as a Local Anæsthetic, Translation by F. Peterson, M.D., . 483
Brooklyn, Treatment of Diphtheria, by Paul H. Kretzschmar, M. D., . . 110
Buffalo Medical Association, . . . 461
Buffalo Medical College, The Thirty-Third Commencement of the . . . 366
Buffalo, Sanitary Condition of, . . . 77

### C.

Cacoepy in Medicine, by J. W. Keene, M. D., . . . . . . . . . . 277
Calomel and Iodide of Potassium in Diseases of the Eye, by Lucien Howe, M. D., (Translation) . . . . . . 352

PAGE.

Calculus Vesical, Spontaneous Discharge of, by Charles G. Stockton, M. D., . 479
Cancer in the Breast, . . . . . . 541
Canula Obstacle to the Final Removal of the After Tracheotomy in Children, 163
Carbolic Acid, Hypodermic Injection of, in the Treatment of Hemorrhoids, . 70
Carbolic Acid Injections for the Cure of Internal Piles, . . . . . . . . 24
Caries of the Ankle in Children, Expectant Treatment in, . . . . . . 497
Cataract, Antiseptic Treatment in Extraction of, . . . . . . . . . 65
Childhood, Hysteria in, . . . . . 160
Chlorate of Potash in the Haemorrhagic Diathesis, . . . . . . . . . 453
Chloride of Ammonium, Treatment of Goitre by, . . . . . . . . . 449
Chloroform Vapor in Earache, . . . 261
Chloroform versus Ether, . . . . . 546
Chloroform, a Death during the Administration of, . . . . . . . . . 531
Cholagogues, . . . . . . . . . 117
Cholera Infantum, . . . . . . 17
Chronic Conjunctivitis, Astringents in, 439
Chronic Purulent Inflammation of the Middle Ear, The effects of, by Lucien Howe, M. D., . . . . . . . . 188
Chrysophanic Acid in Skin Diseases, . 162
Clergymen, Gratuitous Services to, 271, 452
Clinical Reports, . . . . . . . 460
Clinton, George W., LL.D., on Malpractice, . . . . . . . . . 229
Club-foot, Treatment of, By Excision of Bone from the Dorsum Pedis, by Herman Mynter, M. D., (Translation), . 432
Coe, A. L., M.D., on a case of Vesicular Mole, . . . . . . . . . . 250
Cold, Cure for a, . . . . . . . 501
Colic, Treatment of, . . . . . . 265
Colloid Degeneration of the Omentum, by A. G. Crittenden, M. D., . . . 254
Colvin, Darwin, M.D., on Prolapsus of the Ovaries, . . . . . . . 285
Conflagration from Thermo-Cautery during Anæsthesia from Ether, . . 407
Conjunctivitis Gonorrhœal, Transparent Shield by Lucien Howe, M. D., . . 293
Conservative Surgery, a Case of, by J. W. Keene, M. D., . . . . . . 529
Constipation, . . . . . . . . 72

PAGE.

Convergent Strabismus in Children by Mydriotics or Myotics, The treatment of, by Lucien Howe, M.D., (Translated) 255
Cornea, Transplantation of the, . . 66
Coto Bark, . . . . . . . . . 115
Cough Remedy, Oxalate of Cerium as a, 496
Cough Produced by Accumulations in the Ear, . . . . . . . . . 495
Crittenden, A. G., M.D., on Colloid Degeneration of the Omentum, . . . 254
Croup, Tracheotomy for, . . . . 451
Cupro-Sulphate of Ammonia in Neuralgia of the Fifth Pair, by Lucien Howe, M. D., (Translated), . . . . . . 209
Cure for a Cold, . . . . . . . 501
Czerny's Operation for the Radical Cure of Hernia, . . . . . . . . 408

### D.

Death, signs of, in Drowning, . . . 496
Decrease in Bodily Weight after Epileptic Attacks . . . . . . . . 499
Dextro-Quinine, in Periodical Hemicrania, by C. A. Bryce, M. D., . . . 412
Digitalis, in Suppression of Urine External, use of . . . . . . . 29
Digitalis, Iron and . . . . . . 500
Diphtheria, Brooklyn, Treatment of, by Paul H. Kretzschmar, M. D., . . 110
Diphtheritic Poison, The, . . . . . 71
Diphtheritic Croup, Tracheotomy for, . 443
Diphtheria, Benzoate of Soda in, . . 261
Diphtheria, The relation of Membranous Croup to, by J. O. Roe, M. D., . . 325
Diphtheria, Benzoate of Soda in, . . 410
Diplomas, Sale of, . . . . . . 455
Diuretics, Actions of, . . . . . . 544
Druggists' Association, . . . . . 552
Dorr, S. G., M.D., on Injuries to the Cranial Bones, . . . . . . . 475
Duration of Life in the Fœtus in Utero after the Mother's Death, . . . 69

### E.

Earache, Chloroform Vapor in, . 261, 543
Ear, Cough Produced by Accumulations in the, . . . . . . . . . . 495
Eczema Marginatum Iodized Phenol—"Battey's Formula" in, . . . . . 405

PAGE.
Education, Medical, . . . . . . 123
Elevator Employes, Pulmonary Diseases of, by Thos. F. Rochester, M. D., . . 181
Endermic Use of Morphia by "Thimble Blistering," . . . . . . . . 73
Epileptic Attacks, Decrease of Bodily Weight after, . . . . . . . 499
Epilepsy, Thermometer in Trephining for, . . . . . . . . . . 452
Epithetioma of the Cervix Uteri, Removal by Prof. James P. White, M. D., Reported by William D. Granger, M. D., 428
Ergotine Injections, Treatment of Vesical Atony by, . . . . . . . 116
Esmarch's Bandage, Use of Hot Water in Hemorrhage following the Employment of, . . . . . . . . . 116
Ether, The First Insensibility from, . 214
Excision and Amputation of Hip-Joint, 402
Extirpation of the Kidney, by Herman Mynter, M. D., (Translation) . . . 301
Extra-Uterine Pregnancy, Death and Removal of Fœtus, by Prof. James P. White, M. D., Reported by William D. Granger, M. D., . . . . . . 341

### F.

Femur, Luxation of, on Dorsum Ilii, by Wm. C. Phelps, M. D., . . . . . 145
Ferri Mur: Tr., . . . . . . . . 162
Fibrinous Croup, General and Hygienic Treatment of, . . . . . . . 437
Fibro-Cystic Uterine Tumor, Removal of Tumor and Uterus, Reported by William D. Granger, M. D., . . . . . 297
Fibro Sarcoma of the Uterus, by L. C. Lane, M. D., . . . . . . . . 303
Foetus in Utero, Duration of Life in the, After the Mother's Death, . . . 69
Ford, Willis E., M.D., on Hemorrhages from the Lungs, . . . . . . 240
Foreign Bodies on the Iris, by Lucien Howe, M. D., . . . . . . . . 142
Fracture Case, by C. C. F. Gay, M. D., . 351
Fracture Compound Comminuted of the Cranium, . . . . . . . . 431
Fracture of the Neck of the Femur Unrecognized, C. C. Wycoff, M. D., . . 149
Fracture of the Neck of the Femur Unrecognized, by Chas. C. F. Gay, M. D., 98

### G.

PAGE.
Gall, Stone Solvents of, . . . . . 257
Gay, C. C. F., M.D., on Fracture Case, . 351
Gay, Chas. C. F., M.D., on Fracture of the Neck of the Femur unrecognized, . 98
Germany, Medical Expert System in, . 25
Goitre, Treatment of, by Chloride of Ammonium, . . . . . . . . 449
Gonorrhœal Conjuntivitis, Transparent Shield, by Lucien Howe, M. D., . . 293
Gonorrhœa, by Geo. W. Stoner, M. D., . 133
Granger, William D., M.D., report of Extra-Uterine Pregnancy, Death and Removal of Fœtus, . . . . . . 341
Granger, William D., M.D., on Fibro-Cystic Uterine Tumor, removal by Prof. James White, M. D., . . . . 297
Granger, William D., M.D., on a case of the Cervix Uteri, removal by Prof. James P. White, M. D., . . . . . 428
Gratuitous Services to Clergymen, . . 452
Gratuitous Services to Clergymen, . . 271

### H.

Hamilton, Frank H., M.D., on Fracture of the Patella from Muscular Action, . 55
Hæmorrhagic Diathesis, Chlorate of Potash in the, . . . . . . . 453
Hæmarthros, Puncture of, (Translation), by Herman Mynter, M. D., . . . 393
Harvard Medical School, . . . . . 501
Hauenstein, John, M.D., on Placenta Prævia, . . . . . . . . . 103
Health Office of New York, . . . 318
Hemorrhages from the Lungs, by Willis E. Ford, M. D., . . . . . . . 240
Hemorrhage, Post Partum, . . . 397
Hemorrhoids, Treatment of, by Hypodermic Injection of Carbolic Acid, . . 70
Hemorrhoids Treated with Capsicum, . 544
Hepatic Anæmia, . . . . . . 490
Hernia, Czerny's Operation for the Radical Cure of, . . . . . . . . 408
Hiccough Cured by Muriate of Pilocarpine, . . . . . . . . . . 543
High Temperature, Cases of, . . . 543
Hip, How to Grasp the Pelvis for Fixation in contraction of the, by Herman Mynter, M. D., (Translated) . . . 256
Hip-Joint, Amputation of, and Excision, 402

PAGE.

Homœopathic Medicines, a Crucial Test of, . . . . . . . . . . 537
Howe, Lucien, M.D., on the Use of Permanganate of Potassa in Chronic Otorrhœa, . . . . . . . . . 6
Howe, Lucien, M.D., on Foreign Bodies on the Iris, . . . . . . . . 142
Howe, Lucien, M.D., on the Effects of Chronic Purulent Inflammation of the Middle Ear, . . . . . . . 188
Howe, Lucien, M.D., Atrophy of the Optic Nerve in Erysipelas of the Face, (Translation), . . . . . . . 207
Howe, Lucien, M. D., Cupro-Sulphate of Ammonia in Neuralgia of the Fifth Pair (Translation), . . . . . . 209
Howe, Lucien, M. D., the Treatment of Intermittent Convergent Strabismus in Children by Mydriotics or Myotics, (Translated), . . . . . . . 255
Howe, Lucien, M.D., on Sarcomatous Tumors in the Orbit, . . . . . 385
Howe, Lucien, M.D., on Gonorrhœal Conjunctivitis, Transparent Shield, . 293
Howe, Lucien, M.D., on Iodide of Potassium and Calomel in Diseases of the Eye, (Translations) . . . . . . 352
Hospitals in Malpractice Suits, . . . 501
Hot Water, use of, in Hemorrhage following the Employment of Esmarch's Bandage, . . . . . . . . . 115
Hydrate of Chloral, . . . . . . 459
Hydrocele, a New Operation for the Radical Cure of, by Bernard Bartow, M. D., . . . . . . . . . . 527
Hypodermic Injection of Carbolic Acid in the Treatment of Hemorrhoids, . 70
Hypo-Phosphites, Fellows, . . . . 463
Hysteria in Childhood, . . . . . 160
Hysteria in Childhood, by Herman Mynter, M. D., (Translation), . . . 153

I.

Illumination of the Cavities of the Body by a New Instrument, Translated by A. Osterday, M. D., . . . . . . 59
Ingrown Toe-Nail, Treatment of, . . 72
Intra-Uterine Medication, Hints Relative to, by James P. White, M. D., . . 517
Irish Bulls, . . . . . . . . 546
Intravenous Injection of Ammonia, . 61

PAGE.

Injuries to the Cranial Bones, by S. G. Dorr, M. D., . . . . . . . . 475
Insane Asylums, Management of, . . 462
Iodide of Potassium and Calomel in Diseases of the Eye, by Lucien Howe, M. D., (Translation) . . . . . 352
Iodide of Starch, . . . . . . . . 311
Iodized Phenol—"Battey's Formula"—In Eczema Marginatum, . . . . . 405
Iodoform, Solvents of, . . . . . 27
Iron and Digitalis, . . . . . . 500
Iris, Foreign Bodies on the, by Lucien Howe, M. D., . . . . . . . . 142

J.

Jaborandi in the Treatment of Mumps, . 73

K.

Keene, J. W., M.D., on Cacoepy in Medicine, . . . . . . . . . . 277
Keene, J. W., M.D., on A Case of Conservative Surgery, . . . . . . 529
Kidney, Extirpation of the, by Herman Mynter, M. D., (Translation) . . 301
Koosso, A New Method of Administering, 260
Kretzschmar, Paul H., M. D., on Brooklyn Treatment of Diphtheria, . . 110

L.

Lane, L. C., M.D., on Fibro Sarcoma of the Uterus, . . . . . . . . . 303
Laparo Enterotomy Strictura Ilii, Reported by Herman Mynter, M. D., . 346
Lateral Plane Presentation, . . . . 68
Leeches, To Fasten. . . . . . . 28
Leucocythemia, Treatment of, . . . . 489
Long Bones, Osteo Myelitis of the, . 309
Lothrop, Thomas, M.D., on Imperforated Anus, . . . . . . . . . . 151
Lothrop, Thomas, M.D., on Antisepsis in Midwifery, . . . . . . . 41
Lungs, Hemorrhages from the, by Willis E. Ford, M. D., . . . . . . . . 240
Lungs, Oedema of the, . . . . . 539
Luxation of Femur on Dorsum Ilii, by Wm. C. Phelps, M. D., . . . . . 145

M.

Malaria, Vehicle of, . . . . . . 214
Malpractice, by George W. Clinton, L. L. D., . . . . . . . . . . 229
Malpractice Suits, Hospitals in, . . 501

PAGE.

Maltine, . . . . . . . . . 222, 368
Mammary Abscess, . . . . . . 263
Medical Bill, the New, . . . . 548
Medical College, . . . . . . . 172
Medical Education, . . . . . . 123
Medical Expert System in Germany, . 25
Medical Experts, Testimony of, by Hon. Chas. Beckwith, . . . . . . 421
Medical Journalism, . . . . . . 316
Medicine, Cacoepy in, by J. W. Keene, M. D., . . . . . . . . . . 277
Membranous Croup to Diphtheria, The Relation of, by J. O. Roe, M. D., . . 325
Mercury, a New Theory of the Action of, 534
Merriam, E. D., M.D., on Perityphlitic Abscess Treated with Aspiration through Rectum, . . . . . . 205
Metric System, . . . . . . . 318
Middle Ear, The effects of Chronic Purulent Inflammation of the, by Lucien Howe, M. D., . . . . . . . 188
Midwives, . . . . . . . . . 169
Midwifery, Antisepsis in, by Thomas Lothrop, M. D., . . . . . . . 41
Milk, Production and Increase of Secretion of, by the Ricinus Communis, . 451
Morphine, Toleration of, . . . . 450
Mortality, The Influence on, of the Antiseptic Treatment in Wounds of the Head, by Herman Mynter, M. D., (Translation) . . . . . . . 159
Mumps, Jaborandi in the Treatment of, 73
Muscular Action, fracture of the Patella from, by Frank H. Hamilton, M. D., . 55
Muscular Action, Dislocation of Vertebra due to, by John A. Wyett, M. D., . 34
Mydriatics or Myotics, The Treatment of Intermittent Convergent Strabismus in Children, by Lucien Howe, M. D., . 255
Mynter, Herman, M.D., on the Influence on Mortality of the Antiseptic Treatment in Wounds of the Head, (Translated) . . . . . . . . . 159
Mynter, Herman, M.D., on Treatment of Club-Foot from Dorsum Pedis, (Translation) . . . . . . . . . 432
Mynter, Herman, M.D., Osteo Sarcoma of the Superior Maxillary Bone, and of its Treatment, (Translation) . . 389
Mynter, Herman, M. D., Puncture of Haemarthros, (Translation) . . . 393

PAGE.

Mynter, Herman, M.D., on Strictura Ilii, Laparo-enterotomy, . . . . . 347
Mynter, Herman, M.D., on Extirpation of the Kidney, . . . . . . 301
Mynter, Herman, M.D., on Strictura Recti Linear Rectotomy, . . . . 299
Mynter, Herman, M.D., on Perityphlitic Abscess, . . . . . . . . 85
Mynter, Herman, M. D., Unanimo Consensa, (Translation), . . . 208
Mynter, Herman, M. D., How to Grasp the Pelvis for Fixation in Contraction of the Hip, (Translation), . . . 256
Mynter, Herman, M. D., Hysteria in Childhood, (Translation), . . . 153
Mynter, Herman, M.D., Newgrowth in the Bladder of a Child, . . . . 9
Mynter, Herman, M.D., Four Cases of Nerve-Stretching, (Translation) . . 158
Mynter, Herman, M.D., on Aneurism of the Brachial Artery, . . . . . 147

### N.

Narcotic, A New, . . . . . . . 162
Nephrotomy, A Case in, . . . . 407
Nerve-Stretching, Four Cases of, (Translated), by Herman Mynter, M. D., . 158
Nerves, Stretching of, . . . . . 487
Nervous Dyspepsia, Translated by P. W. Van Peyma, M. D., . . . . . . 481
Neuralgia of the Fifth Pair, Cupro-Sulphate of Ammonia in, by Lucien Howe, M. D., (Translation), . . . . 209
Neuralgia, The Treatment of, . . . 411
Night Sweats in Phthisis, Remedy for, . 409
Nitrite of Amyl in Ague, . . . . 213
Nitro-Glycerine, . . . . . . . 449
Nitrous Oxide, Death from, . . . 454

### O.

Oesophagotomy, Case of, . . . . 22
Oedema of the Lungs, . . . . . 539
Omentum, Colloid Degeneration of the, by A. G. Crittenden, M. D., . . . 244
Opium, Belladonna in Poisoning by, . 264
Optic Nerve, Atrophy of the, in Erysipelas of the Face, by Lucien Howe, M. D., . . . . . . . . . 207
Orbit, Sarcomatous Tumor in the, by Lucien Howe, M. D., . . . . . 385
Osteo-Myelitis of the Long Bones . . 309

PAGE.

Osterday, A., M.D., [on Illumination of the Cavities of the Body by a New Instrument, (Translation) . . . . 59
Osteo-Sarcoma of the Superior Maxillary Bone, and of its Treatment, by Herman Mynter, M. D., (Translation) . . . 389
Otorrhœa Chronic, use of Permanganate of Potassa in, by Lucien Howe, M. D., 6
Ovaries, Prolapsus of the, by Darwin Colvin, M. D., . . . . . . . 285
Oxalate of Cerium as a Cough Remedy, . 496
Oxalate of Cerium in Pertussis, . . 116

**P.**

Paper-and-Glue Brace, Angular Curvature of the Spine Treated by the, by Bernard Bartow, M. D., . . . . 202
Paper Spinal Brace, . . . . . . 114
Patella, Fracture of the, from Muscular Action, by Frank H. Hamilton, M. D., 55
Peculiarities of Ringworm and its Treatment, . . . . . . . . . . 355
Pelvis, How to grasp the, for Fixation in Contraction of the Hip, by Herman Mynter, M. D., (Translated) . . . 256
Perineosinuexeriemator, An account of the, by Jacques, Robinson, A. M., M. D., . . . . . . . . . . 30
Perityphlitic Abscess, by Herman Mynter, M. D., . . . . . . . . 85
Perityphlitic Abscess Treated with Aspiration through Rectum, by E. D. Merriam, M. D., . . . . . . 205
Permanganate of Potassa in Chronic Otorrhoea, by Lucien Howe, M. D., . 6
Periodical Hemicrania, Dextro-Quinine in, . . . . . . . . . . .
Peterson, F., M.D., on Bromide of Ethyl as a Local Anæsthetic, (Translation), . 483
Peterson, Fred, M.D., Report of Traumatic Aneurism, . . . . . . 199
Per-Vaginal Enucleation of the Uterus, 502
Phelps, Wm. C., M.D., on Luxation of Femur on Dorsum Ilii, . . . . 145
Phthisical Cough, The Treatment of . 360
Phthisis, Remedy for Night Sweats in, . 409
Piles, Internal, Carbolic Injéctions for the Cure of, . . . . . . . . 24
Pilocarpin in Skin Diseases, . . . 543
Pitt, W. H., M. D., on Spectrum Analysis of Blood, . . . . . . . . 1

PAGE.

Placenta Praevia, by John Hauenstein, M. D., . . . . . . . . . 103
Placenta Praevia, The Method used in Germany in the Treatment of, by Louis Shade, M. D., . . . . . . . 206
Poisoning by Sodium Salicylate, . . 73
Postal Laws and our Subscribers, . . 512
Post Partum Hemorrhage, . . . 397
Pott's Disease, Treatment of . . . 500
Professional Cowardice, . . . . 220
Profits of Druggists, A Shrewed Calculation on the, . . . . . . . 454
Prolapsus of the Ovaries, by Darwin Colvin, M. D., . . . . . . . 285
Pruritis, Balsam of Peru in, . . . 361
Puerperal Cystitis, The Etiology of, . 403
Puerperal Eclampsia, by F. W. Bartlett, M. D., . . . . . . . . . . 248
Pulmonary Diseases of Elevator Employees, by Thos. F. Rochester, M. D., 181
Pulsatilla, The Therapentic Value of, . 448

**R.**

Renal Inadequacy, . . . . . . 394
Rheumatism, Salicylate of Soda in, . . 29
Ricinus Communis, Production and Increase of Secretion of Milk by the, . 451
Ringworm and its Treatment, Peculiarities of, . . . . . . . . . 355
Rochester, Thos. F., M.D., on Pulmonary Diseases of Elevator Employees, . . 181
Rochester, Thomas F., M.D., on State Medicine, . . . . . . . . . 373
Roe, J. O., M.D., on the relation of Membranous Croup to Diphtheria, . . 325
Rupture of the Duodenum from a Blow on the Abdomen, . . . . . 492
Rupture of the Uterus, by P. W. Van Peyma, M. D,, . . . . . . . . 108

**S.**

Salicylate of Soda in Rheumatism, . . 29
Salicylic Acid against Tænia, . . . 27
Salicylic Acid Detrimental to Health, by E. V. Stoddard, M. D., (Translated), . 14
Sanitary Condition of Buffalo, . . . 77
Sarcomatous Tumours in the Orbit, by Lucien Howe, M. D., . . . . . 385
Sarcoma of Left Wrist, . . . . . 430
Schade, Louis, M. D., The Method used in Germany in the Treatment of Placenta Praevia, (Translated) . . . 206

PAGE.

Scott, Preston, M.D., on Cholera Infantum, . . . . . . . . . . 17
Shield, Transparent, in Gonorrhœal Conjunctivitis, by Lucien Howe, M. D., . 293
Skin Diseases, Chrysophanic Acid in, . 162
Slacer, William H., M.D., on Remarkable Vitality, . . . . . . . 107
Sodium Salicylate, Poisoning by, . . 73
Solvents of Gall-Stones, . . . . 257
Spectrum, Analysis of Blood, by W. H. Pitt, M. D., . . . . . . . 1
Sphygmograph for Diagnosis of Peculiary Slow Pulse, . . . . . . . 24
Spinal Brace Paper, . . . . . . 114
Splenotony, . . . . . . . . . 209
State Medicine, by Thomas F. Rochester, M. D., . . . . . . . . . . 373
Stockton, Charles G., M.D., on Spontaneous Discharge of a Vesical Calculus, . 479
Stoddard, E. V., M.D., Salicylic Acid Detrimental to Health, . . . . . 14
Stoner, Geo. W., M.D., on Gonorrhea, . 133
Stretching of Nerves, . . . . . . 487
Strictura Recti Linear Rectotomy, Reported by Herman Mynter, M. D., . 299
Superior Maxillary Bone, Ostero-Sarcoma of the, and of its treatment, by Herman Mynter, M. D., (Translation), 389
Syphilis, "Tonic Treatment" of, by P. W. Van Peyma, M. D., . . . . . . 290
Syphilis by Vaccination, . . . . . 117

**T.**

Taenia, Salicylic Acid against, . . . . 27
Testimony of Medical Experts, by Hon. Chas. Beckwith, . . . . . . . 469
Thermometer in Trephining for Epilepsy 452
"Thimble Blistering," Endermic use of Morphia by, . . . . . . . . 73
Toe Nail, Ingrown, Treatment of, . . 72
"Tonic Treatment" of Syphilis, by P. W. Van Peyma, M. D., . . . . . 290
Tracheotomy for Diphtheritic Croup, . 443
Tracheotomy for Croup, . . . . . 451
Transplantation of the Cornea, . . 66
Traumatic Tetanus, Atropia in, . . 308
Twin Labors, . . . . . . . . 67
Two Points in the History of a Case, . 509

**U.**

University of Buffalo, . . . . . . 363
Unanimo Consensu, by Herman Mynter, M. D., . . . . . . . . . . 208
Urine, External Use of Digitalis in Suppression of, . . . . . . . . 29
Urine, Retention of the, . . . . . 26
Uterine Hemorrhage, Auscultation in, . 164
Uterus, Rupture of the, by P. W. Van Peyma, M. D., . . . . . . . 108
Uterus, Per-Vaginal Enucleation of the, 502
Uterus Torn from a Puerperal Woman, . 498

**V.**

Vaccination Syphilis, . . . . . . 117
Vaccinium Crassifolium Aut Repens, . 498
Van Peyma, P. W., M. D., Nervous Dyspepsia, (Translation) . . . 481
Van Peyma, P. W., M.D., on Rupture of the Uterus, . . . . . . . . 108
Van Peyma, P. W., M.D., on "Tonic Treatment" of Syphylis, . . . . 290
Vertebra, Dislocation of, due to Muscular Action, by John A. Wyett, M. D., . 34
Vesical Atomy, Treatment of, by Ergotine Injections, . . . . . . . 116
Vesicular Mole, A case of, by A. L. Coe, M. D., . . . . - . . . . . 250
Vinegar as a Post Partum Hemostatic, . 215
Vitality, Remarkable, by William H. Slacer, M. D., . . . . . . . 107
Vivisection, . . . . . . . . . 319
Vital Statistics of Buffalo, . . . . 365

**W.**

White, Prof. James P., M.D., Case of Epithelioma of the Cervix Uteri, Reported by William D. Granger, M.D., 428
White, Prof. James P., M.D., Fibro-Cyrtic Uterine Tumor, Removal of Tumor and Uterus, Reported by William D. Granger, M. D., . . . . .
White, Prof. James P., M.D., Extra Uterine Pregnancy; Death and Removal of Foetus, . . . . . . 341
White, James P., M.D., on Hints Relative to Intra-Uterine Medication, . . 519
Women as Physicians, . . . . 409
Whooping Cough by Atropia, Treatment of, . . . . . . . . . . 357
Whooping Cough, . . . . . . 28
Wyckoff, C. C., M.D., on an Unrecognized Fracture of the Neck of the Femur, . . . . . . . . . . 149
Wyett, John A., M.D., on Dislocation of Vertebra due to Muscular Action, . 34

**Z.**

Zinc, Phosphide of, . . . . . . 259

BOOKS AND PAMPHLETS RECEIVED.

**Manual of the Principles and Practice of the Operative Surgery**. By STEPHEN SMITH, A. M., M. D. Surgeon to Bellevue and St. Vincent Hospitals, New York. Boston: Houghton, Osgood & Co.

**Transactions of the State Medical Society of Arkansas at its Fourth Annual Session.**

**Thevetia Iccotli and its Glucoside**. By DAVID CERNA, M. D.

**The Treatment of Epithelioma of the Cervix Uteri**. By J. MARION SIMS, M. D.

**The Popular Science Monthly,** conducted by E. L. and W. J. YOUMANS.

**A Clinical Treatise on the Diseases of the Nervous System**. By M. ROSENTHAL, Professor of diseases of the Nervous System at Vienna. Translated by L. Putzil, M. D. New York: William Wood & Co.

